# A Review on the Use of the HPV Vaccine in the Prevention of Cervical Cancer

**DOI:** 10.7759/cureus.28710

**Published:** 2022-09-02

**Authors:** Pranav Pathak, Sandhya Pajai, Himanshi Kesharwani

**Affiliations:** 1 Obstetrics and Gynaecology, Jawaharlal Nehru Medical College, Datta Meghe Institute of Medical Sciences, Wardha, IND

**Keywords:** womens health, cervical cancer prevention, cancer cervical, cervical cancer vaccines, gardasil, immunoprevention, vaccination, human papillomavirus

## Abstract

The main risk factor for invasive cervical carcinoma is persistent infection by the high-risk human papillomavirus (HPV). HPV is the most prevalent sexually transmitted infection (STI) and has been linked to 15 different cancers. Cervical cancer is one of the most frequent cancers among women, particularly in resource-limited countries. Cervical cancer is an HPV disease with the highest worldwide burden in resource-limited nations. With improved medical care and nationwide screening programmes, the mortality rate from cervical cancer has decreased in the past 40 years. Many developing nations have been shown to have inadequate knowledge and health-seeking practices, making proper awareness and immunisation programmes necessary. The best strategy to reduce the incidence of cervical cancer is through the administration of HPV vaccines along with routine cervical screening. The HPV vaccine is crucial for public health. Vaccinations against all HPV subtypes, namely, bivalent, quadrivalent, and nonavalent, are available. Financial issues are the main barrier to HPV vaccination. The framework for behavioural and social drivers of vaccination, which includes practical concerns, motivation, social processes, thoughts, and feelings, is widely used to uncover important aspects linked with HPV vaccination. The burden of cervical cancer due to HPV and the advantages of HPV vaccination are summarised in this review article.

## Introduction and background

The human papillomavirus (HPV) belongs to the Papillomaviridiae family and is a DNA virus most commonly implicated in causing sexually transmitted diseases. Until now, more than 40 HPV subtypes have been isolated that have the potential to cause infection in the genital areas of both genders. Different subtypes of the human papillomavirus affect different areas of our body. But among all the subtypes, a total of only 15 human papillomavirus subtypes are related to the development of cervical cancer. Among the subtypes causing cervical cancer, the HPV 16 genotype accounts for the causative organism in 70% of cases [1].

Risk factors for HPV

The contribution of risk factors like alcohol consumption, cigarette smoking, exposure to the sun, and other radiation for a prolonged period to the causation of cancer is well-documented. But the general public is less aware that a significant amount of the world's cancer burden is linked to infectious diseases [2]. Epidemiological studies suggest that socioeconomic factors like education and income, sexual and reproductive factors, and other lifestyle factors and specific health behaviours mentioned above contribute to the development of cervical cancer [3]. HPV 16, which belongs to the high-risk HPV group, is a major risk factor in developing invasive cervical cancer. Therefore, there could be a significant decrease in the incidence of cervical cancer by preventing persistent HPV infection. This can be achieved through health care promotion, education, and counselling about safe sexual practices, delaying first intercourse, male circumcision, and early diagnosis and treatment of any vaginal infection [4].

The burden of HPV

**Figure 1:**
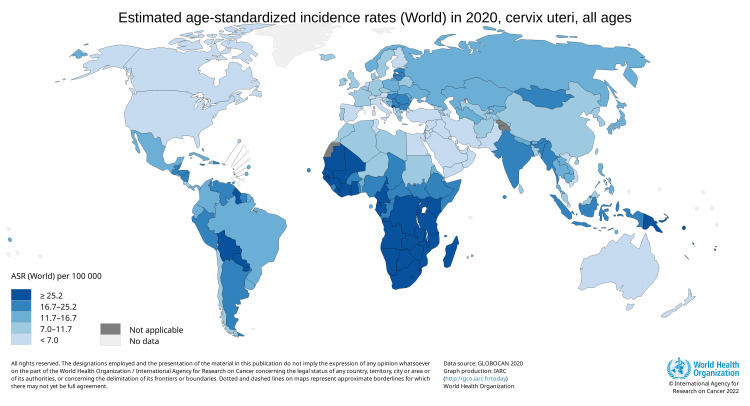
Estimated age-standardized incidence rates (World) in 2020, cervix uteri, females, all ages Source: GLOBOCAN 2020

**Figure 2:**
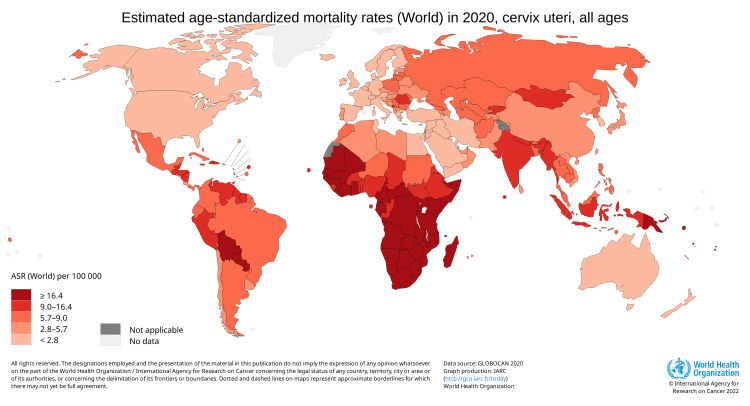
Estimated age-standardized mortality rates (World) in 2020, cervix uteri, females, all ages Source: GLOBOCAN 2020

The incidence of cervical cancer is highest among 50-to 55-year-old females, and the highest mortality is seen in females aged 75 years and over. This suggests that there is a slow progression of cancer after acquiring an infection at a younger age [5]. Low socioeconomic status is among the important risk factors for developing cervical cancer. This is reflected by the fact that around 85% of all cervical cancer cases are reported in underdeveloped countries [4]. India, where 16-17 per cent of the world's women live, is home to 27 per cent of all cervical cancer incidences worldwide [6]. Among India's 60,000 annual deaths, nearly 33 per cent of all cervical cancer deaths worldwide are attributed to India, along with 100,000 newly diagnosed cases [7]. According to Globocan 2020, there were 6,04,100 new cases found worldwide in 2020, and cervical cancer was responsible for 341,831 deaths, as shown in Figure 1 and Figure 2.

In 2020, cervical cancer accounted for 18.3 per cent (123,907) and 9.4 per cent of all cancer cases in India. It is one of the more prevalent malignancies in India and is the main factor in the death rate from cancer among women in underdeveloped nations [8,9]. Therefore, cancer prevention techniques are required to lessen financial strain and human suffering. In addition to being a part of tertiary prevention for cancer patients who have already had curative therapy to prevent a recurrence, vaccinations are a part of primary prevention to lower the incidence of premalignant and malignant cancer [10]. A target for eliminating cervical cancer globally has been set by the World Health Organization (WHO), which is defined as having an annual incidence of less than 4 per 100,000 women [11]. Three crucial pillars and their accompanying goals are necessary to achieve that target. They are:

Vaccination - 90% of girls are fully vaccinated with the HPV vaccine by the age of 15; Screening: 70% of women are screened using a high-performance test by the age of 35 and again by the age of 45; Treatment: 90% of women with pre-cancer are treated, and 90% of women with invasive cancer are managed.

Reaching the 90-70-90 target by 2030 will put each country on the path to eradicating cervical cancer within the next century [12].

The National Cancer Control Programme in India

The National Cancer Control Programme (NCCP) was established in 1975 to provide the best cancer hospitals and institutes with the appropriate equipment to treat the disease. The national programme was altered in 1984, with a greater emphasis placed on the primary prevention of disease occurrence and early cancer case identification. The agenda was further expanded in particular districts with medical college hospitals from 1990-1991 and the District Cancer Control Programme was launched. In the 2000s, it experienced additional alterations. The National Programme for Prevention and Control of Cancer, Diabetes, Cardiovascular Illnesses, and Stroke (NPCDCS) project, initiated in 2010 to prevent and control serious non-communicable diseases, now includes cancer control [13].

## Review

HPV vaccination

A) Vaccination as a Tool to Prevent Cancer

In India, asymptomatic female screening is seldom done. Hence, immunisation against HPV is a promising method of preventing cervical cancer. Both HPV vaccines, Cervarix and Gardasil, are equally effective against recurrent HPV 16 and 18 infections [4]. HPV vaccination offers primary prevention against HPV types 16 and 18 [14,15]. The dissemination of the vaccine to underdeveloped nations has been uneven, even though vaccination with Cervarix and now, Gardasil, has demonstrated its effectiveness in the immunoprevention of cervical cancer. Vaccine uptake has encountered several difficulties, even in nations where the vaccine is widely available [16].

B) Vaccines Available in India

Since 2007, four prophylactic vaccines, namely, Gardasil, Cervarix, Gardasil 9, and Cecolin, have been approved for use against HPV infection. As shown in Table 1, the number of HPV strains each vaccine is designed to prevent varies. As a result, not all HPV-related cancers can be avoided by the current vaccines [2].

**Table 1:** HPV vaccines available in India and the HPV strains each vaccine is designed to target * Licensed in China in 2020 and currently under review by the World Health Organization [17]

Brand name	Gardasil	Cervarix	Gardasil 9	Cecolin
Developer	Merck	GlaxoSmithKline	Merck	Xiamen Innovax Biotech
Date licensed	2006	2007	2014	2020*
Type of vaccine	Quadrivalent	Bivalent	Nonavalent	Bivalent
HPV strains targeted	6, 11, 6, 18	16, 18	6, 11, 16, 18, 31, 33, 45, 52, 58	16, 18

The first commercially available vaccinations to prevent HPV infection were Cervarix, a bivalent HPV vaccine that protects against two subtypes of HPV, and Gardasil, a quadrivalent HPV vaccine that targets four subtypes of HPV, as given in the above table. The Gardasil 9 vaccine, commonly known as the nonavalent HPV vaccine, provides defence against both high-risk and low-risk HPV strains [18]. The vaccines provide long-term protection against primary HPV vaccine-type infection and a minimal level of cross-protection, and they seem to be safe [19]. The HPV vaccine is crucial for public health. In India, there is little compliance with cervical Pap smear screening. The vaccines that are currently available are reliable and effective. The HPV vaccine is now widely accepted and a part of immunisation programmes in many nations. The vaccine should be given before the first sexual encounter because protection occurs only when given before HPV infection. Ideally, parents should first learn about the vaccine as a cervical cancer prevention vaccine rather than a sexually transmitted disease vaccine [20].

C) Vaccine Dose and Schedule

The 0.5 mL intramuscular dose, which can be given in either the anterolateral thigh or deltoid muscle, is available as a single-dose vial of sterile suspension for injection or as a prefilled syringe, both of which need to be thoroughly shaken before use. It is important to adhere to the manufacturer's recommendations for vaccine storage and administration [21]. The WHO's Strategic Advisory Group of Experts on Immunization (SAGE) concluded that the mounting scientific evidence shows single-dose schedules are just as effective as two- or three-dose regimens. The vaccine offers reliable protection against HPV, the virus that causes cancer of the cervix, and is comparable to 2-dose schedules, according to SAGE's evaluation.

SAGE suggests changing HPV dosage schedules as follows: One or two doses for the primary target of girls aged 9-14; one or two doses for young women aged 15-20; two doses separated by six months for women over the age of 21. If possible, immunocompromised people, including those with HIV, should receive three doses; if not, they should receive at least two doses [22]. The first dose must be given between 9 and 12 years of age and should be completed before 26 years of age. The hepatitis B and tetanus, diphtheria, and pertussis (Tdap) vaccines can be administered alongside the HPV vaccine [23,24].

D) The Mechanism of Vaccine Action

Given that HPV exclusively affects humans, it is difficult to determine how the nonavalent HPV vaccination works. However, it is thought that the humoral response is what causes the vaccination to work. Nonavalent HPV vaccines are created using the L1 virus-like particles (VLP) of the carcinogenic protein subunit (CPS) component of the HPV types 6, 11, 16, 18, 31, 33, 45, 52, and 58. According to 2016 immunogenicity research, the vaccine's inactive HPV L1 virus-like particles (VLPs) generate antibodies that neutralise diverse HPV strains and trigger a potent humoral immune response that protects against illnesses and dysplastic lesions brought on by HPV. According to the same study, the nonavalent HPV vaccine raised antibody titers that are 10-100 times higher than those brought on by naturally occurring infection. The effectiveness of the vaccine, therefore, seems to be mediated by humoral response mechanisms [25].

Issues with HPV vaccination in India

For nations like India, which have a high disease burden, HPV vaccinations can be a game-changer for cancer prevention. According to the WHO's position paper on the subject, the vaccine must be integrated into national immunisation programmes worldwide [21]. Despite the benefits of HPV vaccination, herd immunity cannot be achieved globally due to the current level of vaccine uptake. Models indicate that eliminating HPV infections will require worldwide vaccination rates of 80% to achieve the WHO Cervical Cancer Elimination Strategy's goal of promoting HPV vaccination to 90% of all adolescent girls by 2030 [2]. However, the average completion rates for all WHO areas in 2020 ranged from 29% to 60% [26].

A) Health Priorities and Vaccine Cost

Although the development of HPV vaccines has raised expectations for the future elimination of cervical cancer, the inclusion of HPV vaccination in India's immunisation programme has been hotly contested. The government's programme's inclusion of the HPV vaccine may not be at the top of the list of health priorities for India. Given that the public sector's spending on health is extremely high, without assistance from outside, it is challenging for the Indian government to incorporate a costly vaccine into the country's national vaccination programme [27].

B) Epidemiological Evidence on Cervical Cancer

Various Indian regions are underrepresented in data from overlapping sources such as the National Cancer Registry Programme (NCPR) reports, Cancer Atlas, C15, and Globocan cancer data [28]. According to a recent analysis of cancer epidemiology in India, cervical cancer, which has a burden of 17.1 per cent among females aged 30-69, is the most prevalent deadly malignancy [29]. According to another study, the age-adjusted mortality rate for cervical cancer was 7.7 per 100,000 [28]. According to earlier research, the death rate in rural areas is approximately 65.5 [30]. The epidemiological statistics from India regarding HPV and cervical cancer are inconsistent. Due to the wide range of estimates for the illness burden, which ranges from a low of 7.7 to a high of 65.5, policymakers confront significant hurdles in measuring the real disease burden for the implementation of the cervical cancer vaccination programme [4].

C) Vaccine Acceptance

According to a survey, 72% of educated urban males and females in the middle or upper socioeconomic levels who had at least one girl child had never heard of HPV. After reading a brief information sheet regarding the HPV vaccine, 80% of parents stated they would vaccinate their daughters, even though only 46% of parents intended to protect their girls against STIs [31].

D) Vaccines and Cervical Cancer Screening

Vaccination against HPV is as important as cervical cancer screening. There are several obstacles to overcome to effectively employ the vaccine to control this totally avoidable disease. These include lowering vaccine costs, receiving government and policymaker support, educating at all levels, and removing immunisation barriers. The ideal plan for prevention should include the immunisation of teenagers before the onset of sexual activity and screening for and treating precancerous lesions, a surrogate marker for cervical cancer [4].

Factors that can promote HPV Vaccine coverage

A) Raising Public Awareness

It is important to promote the integration of cancer prevention and education into every parent and caregiver's routine and to develop a plan for the long-term monitoring of girls who have received the vaccine. It is necessary to identify the gaps and hurdles to access and delivery of HPV vaccination. The best way to influence and increase public awareness is through communication. It is crucial to inform parents and caregivers about the cervical cancer vaccine and to develop uplifting, intelligible messages that the public and the media can recognise as their own. The government's spending on cancer treatment facilities in India is very high; this, in turn, has a positive economic impact [20].

B) Impact of Education

The findings of the study showed that parents had a limited grasp of the health issues linked to HPV infection, its prevention, and modes of transmission. The HPV vaccine is readily available, and the value of taking precautions and its advantages in preventing cancer was unknown to nearly half the subject population. However, 43.75% of the respondents said they would be open to receiving an HPV vaccination. The study's findings indicated a substantial knowledge gap between before and after the intervention, but there was no appreciable change in perception. Although there is a minimally discernible change in awareness, a constructive behavioural change was seen in favour of vaccination, which might eventually influence the subject's perception. Our study and other outreach initiatives would be crucial in reducing the prevalence of genital cancer worldwide. The research even suggested that school can be a good place to educate and communicate with teenage females about getting vaccinated against HPV [32].

C) Measuring Behavioural and Social Drivers of Vaccination

The WHO formed the "Measuring Behavioural and Social Drivers of Vaccination" (BeSD) working group in 2018 to better identify the factors that influence vaccine coverage. A team of global health specialists created the BeSD framework to collect and utilise information on the behavioural and social factors that influence vaccination. The framework comprises four areas that can be changed to influence vaccine uptake [33-36]. They are practical issues, motivation, social processes, and thoughts and feelings. 

## Conclusions

Given the wide range and seriousness of illnesses brought on by infection, addressing HPV and its associated pathologies will remain a top priority in public health. Despite the advancement of immunisation for illness prevention, there is still a sizable worldwide burden from these disorders. Many benign and malignant diseases, as well as HPV infection, are possible outcomes. The HPV vaccine's untapped potential to reduce cancer risks, costs, and psychological suffering stands out, as it is the only vaccine that can prevent cancer. In order to provide effective cervical cancer screening programmes and community-level activities to increase community engagement and knowledge about cervical cancer, India must strengthen the capacity of its health system. This can only be accomplished by training a workforce of paramedical and health professionals who are informed about screening. Partnerships must be made with international businesses that make vaccines, diagnostic devices, and cancer therapies in the private sector as part of an immediate, coordinated effort. This is necessary to enable efficient intervention and stop the almost 350,000 deaths from cervical cancer predicted for 2021 and beyond. Vaccination programmes are still being developed and expanded, but public acceptance of vaccination still faces cultural hurdles, necessitating public awareness campaigns to increase vaccination compliance. Despite obstacles to worldwide vaccine distribution, the vaccine's effectiveness and effects on global public health should not be understated. Ongoing efforts are required to optimise the vaccine's benefits for the individual and the entire population. Once these objectives are met, cancer immunoprevention through vaccination will become the norm for long-term cancer treatment.
